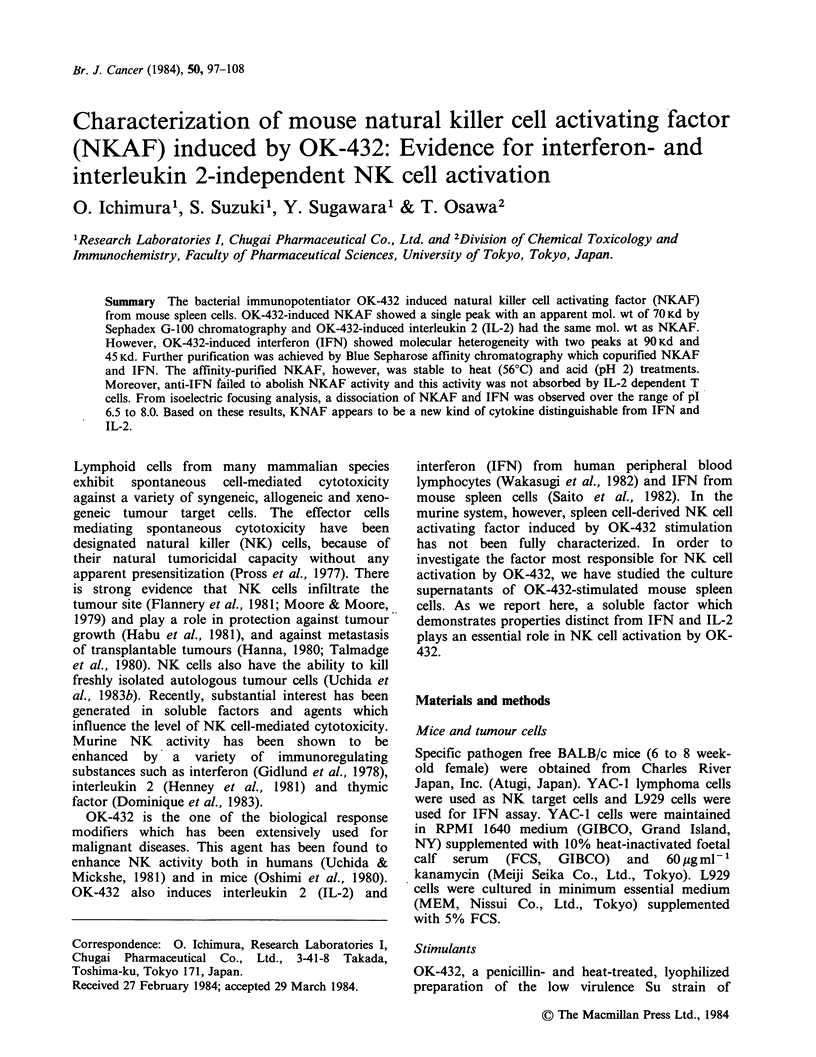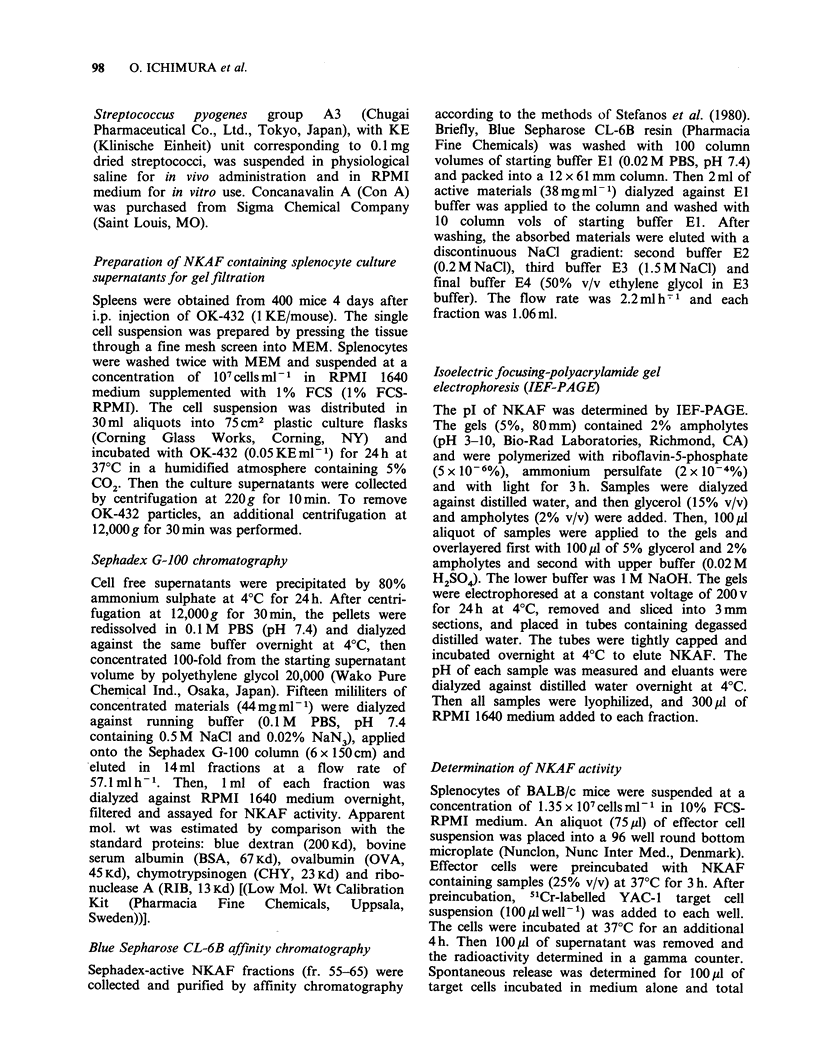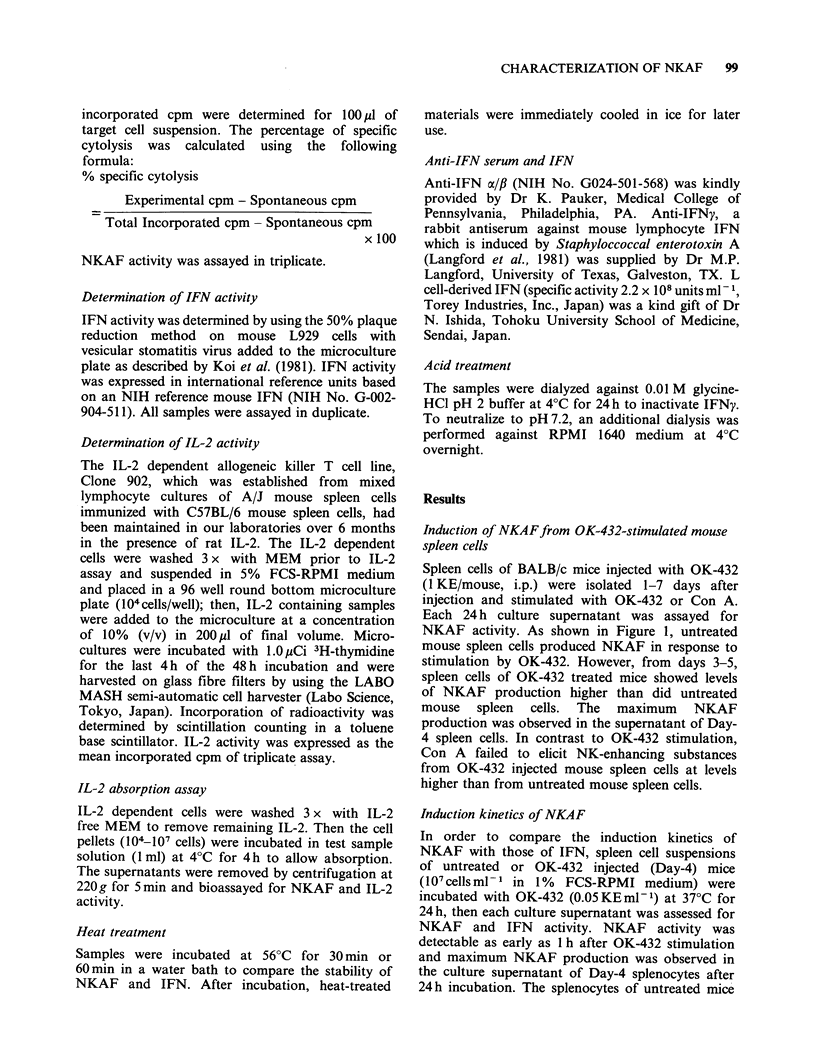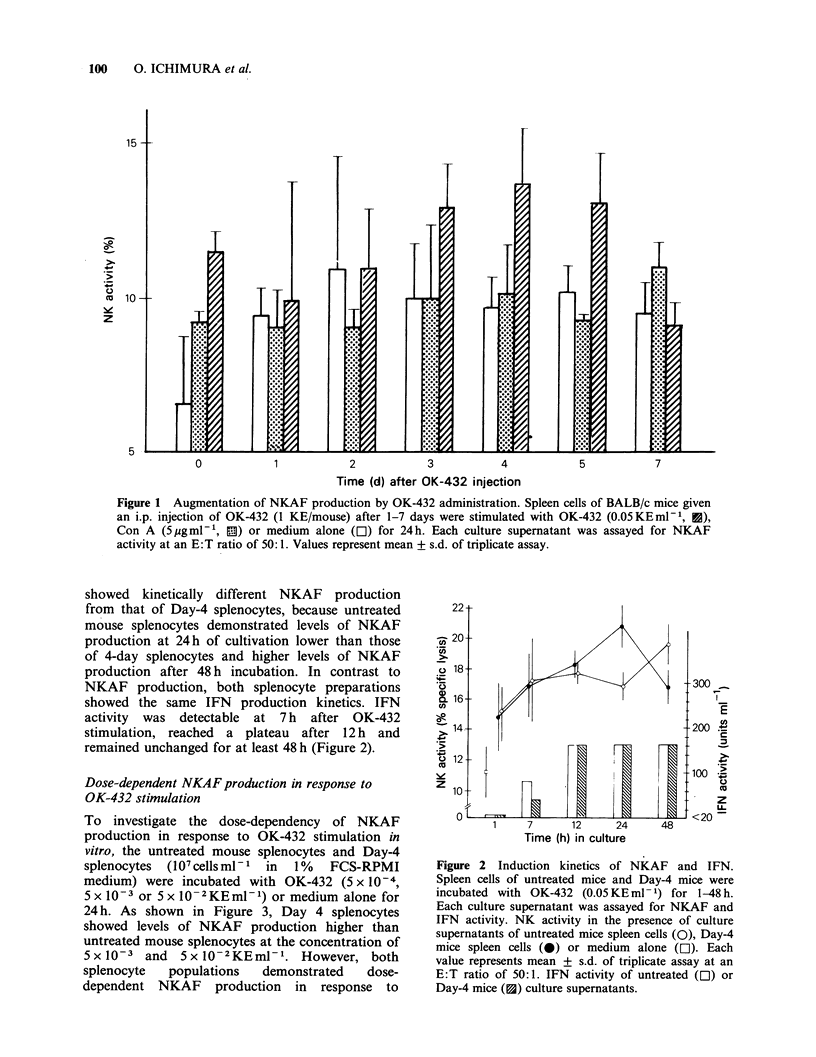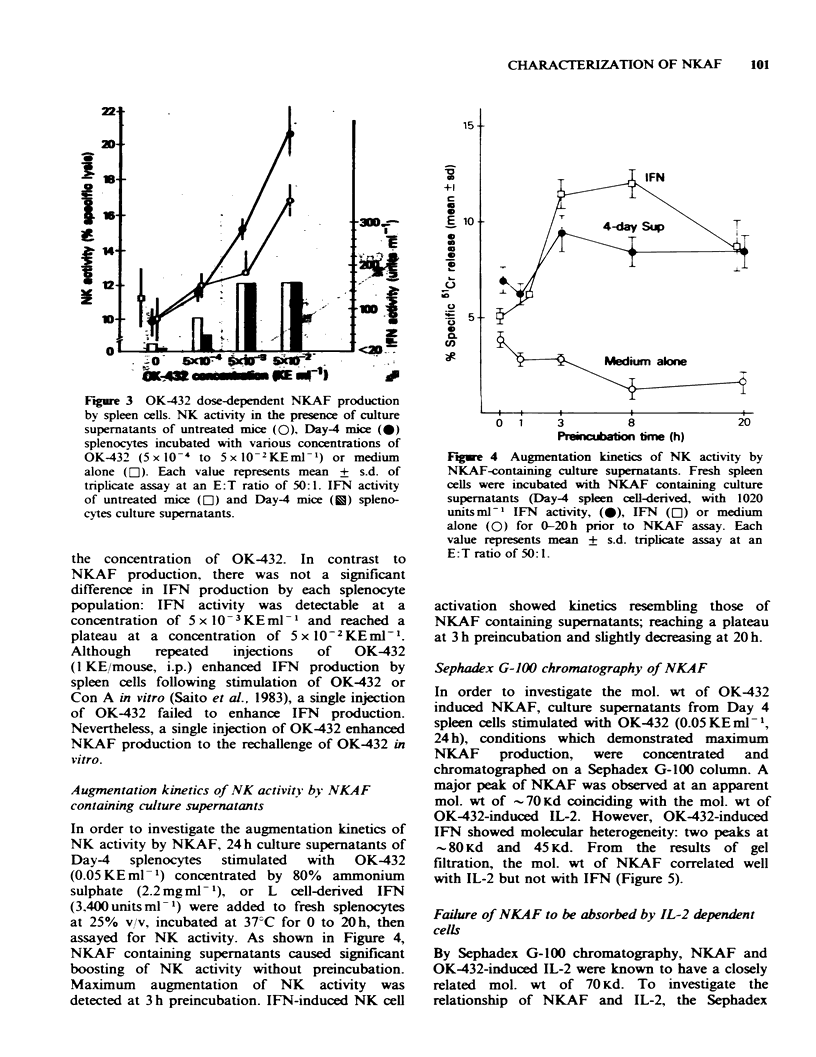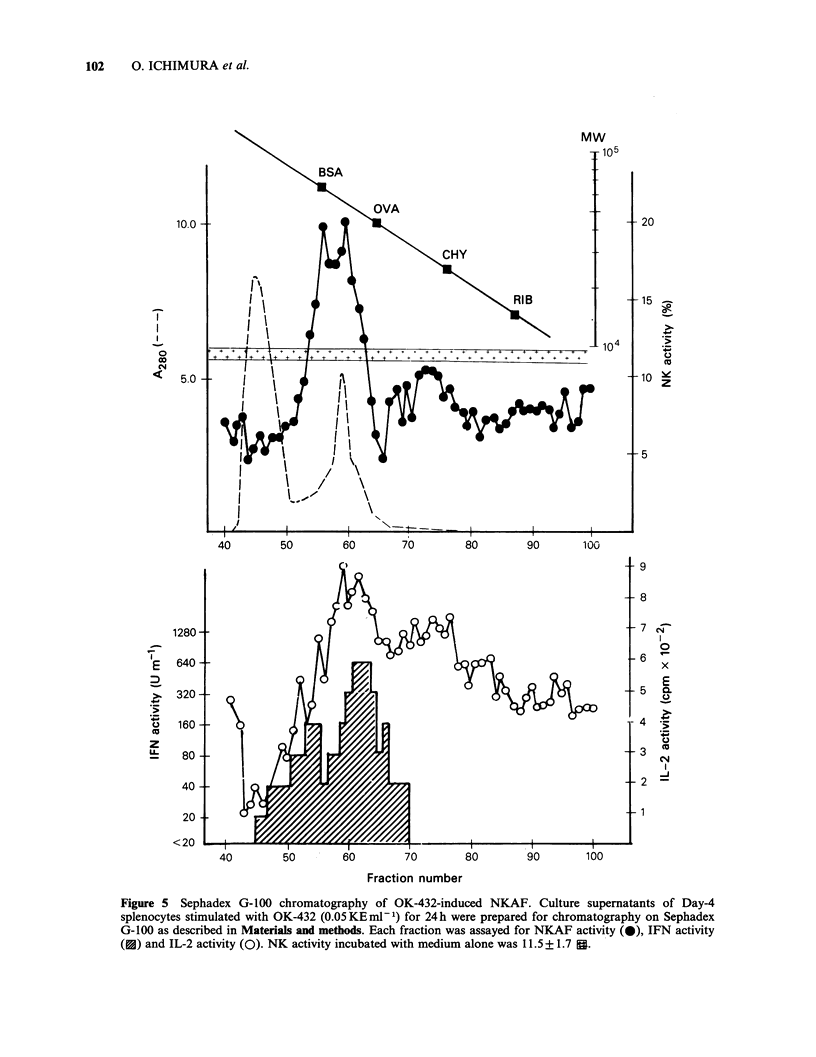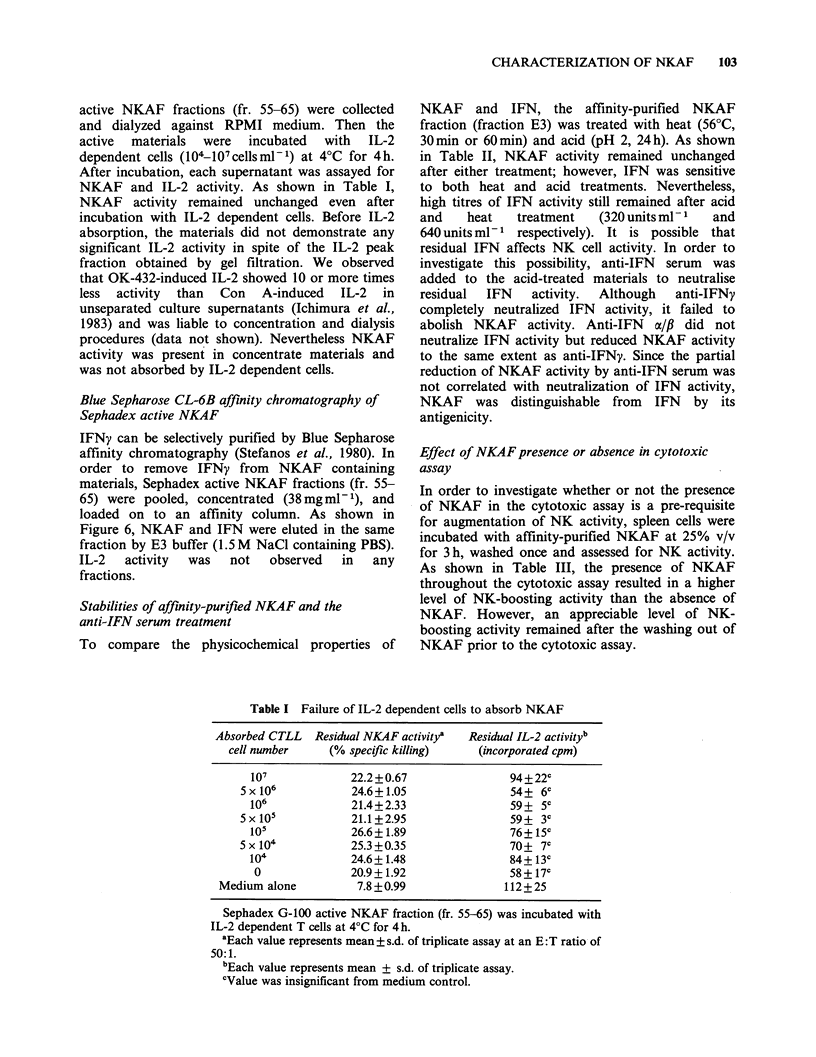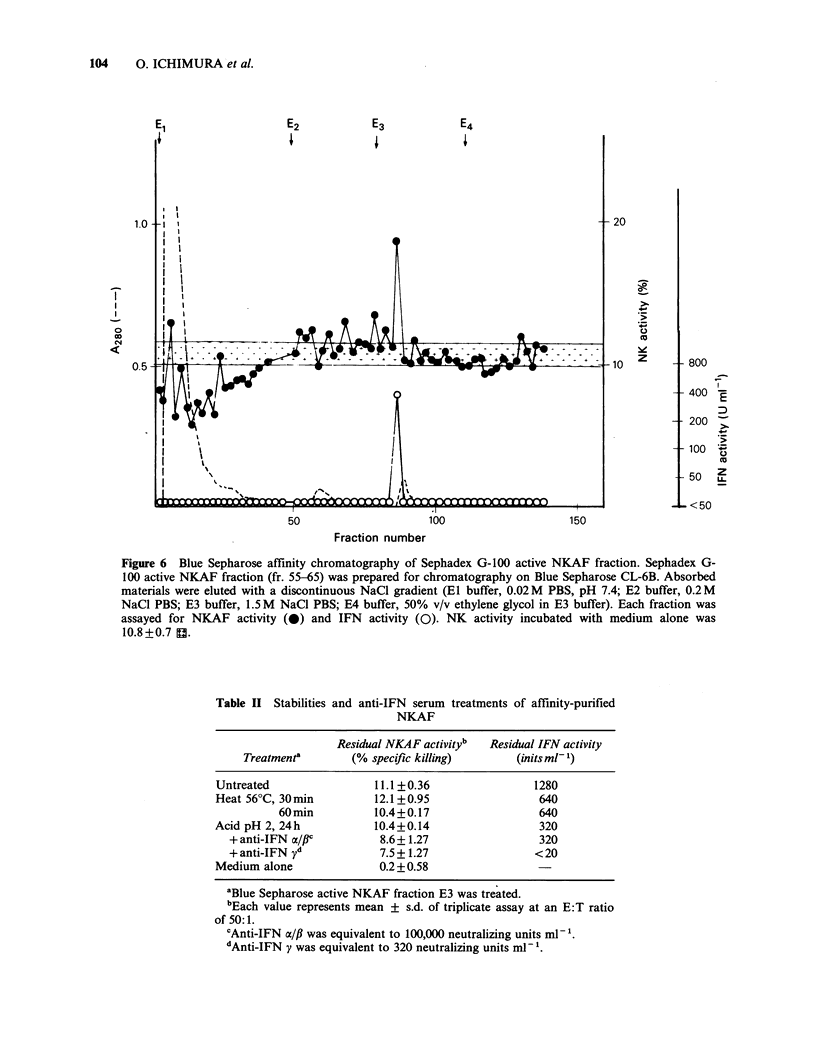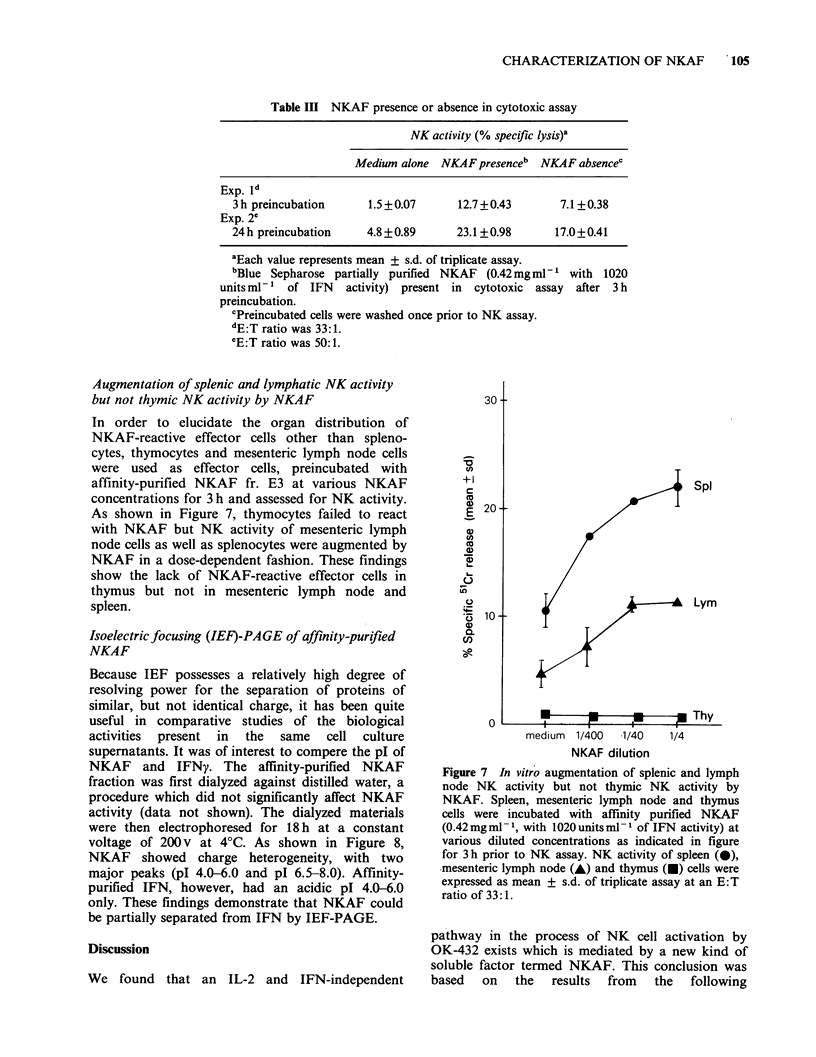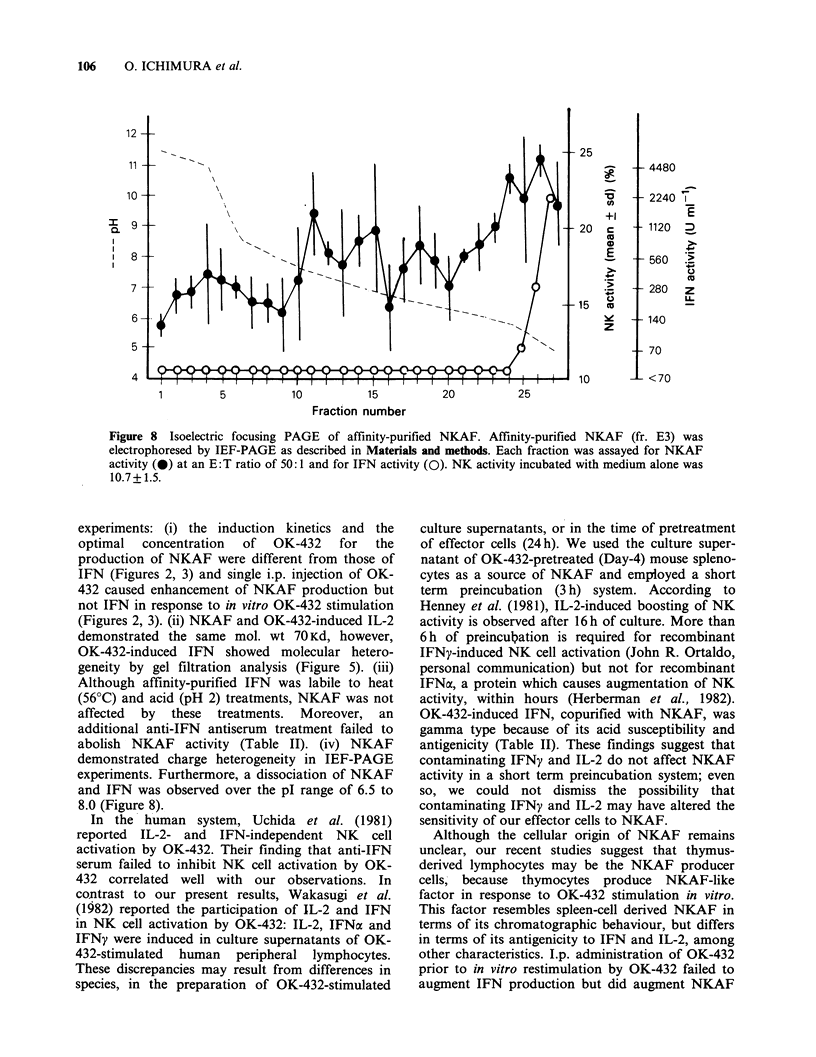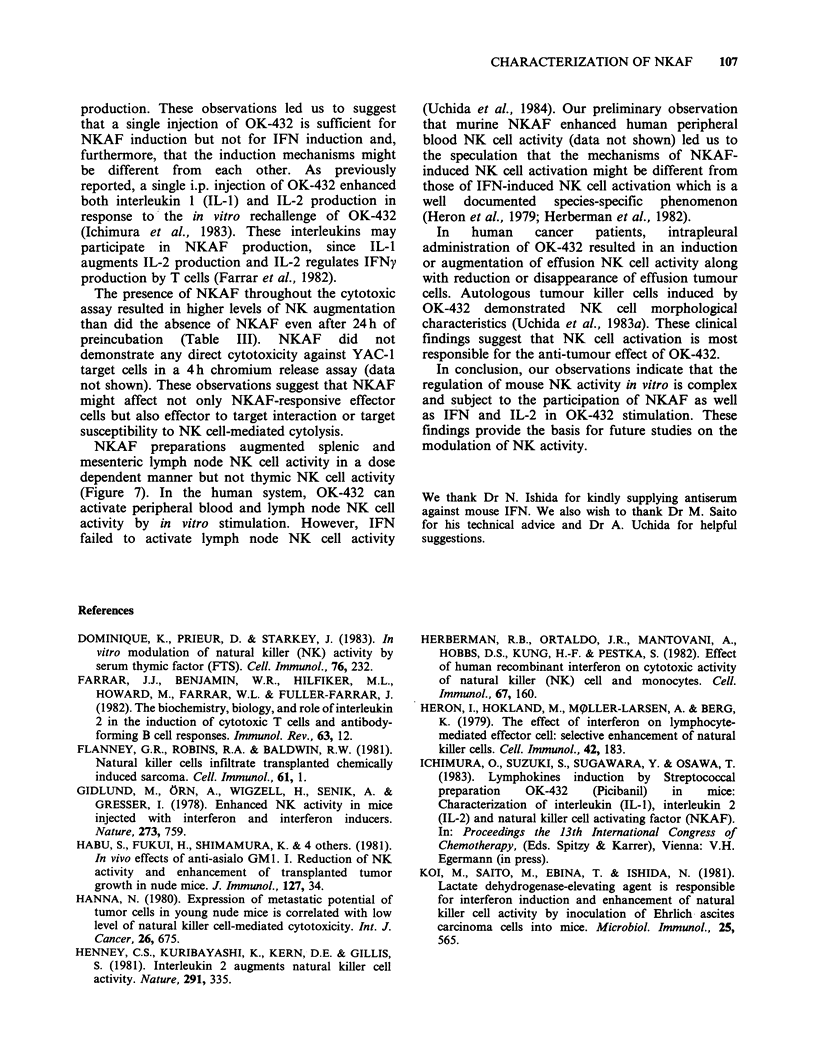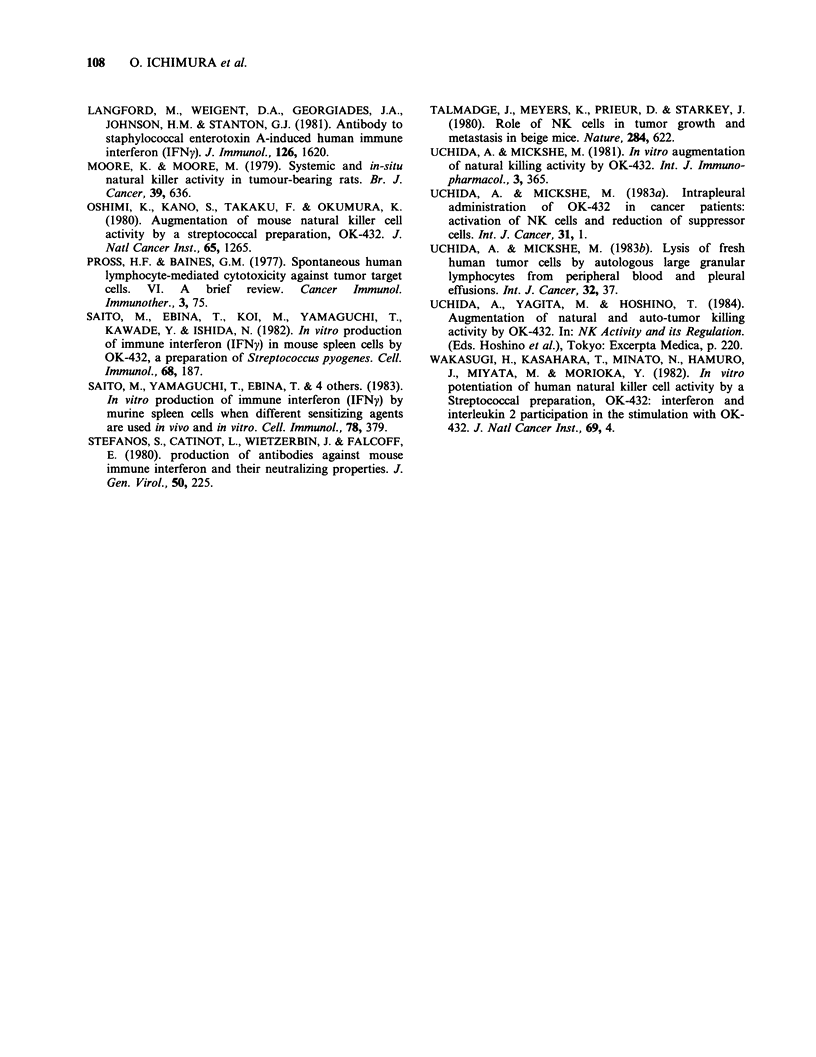# Characterization of mouse natural killer cell activating factor (NKAF) induced by OK-432: evidence for interferon- and interleukin 2-independent NK cell activation.

**DOI:** 10.1038/bjc.1984.144

**Published:** 1984-07

**Authors:** O. Ichimura, S. Suzuki, Y. Sugawara, T. Osawa

## Abstract

The bacterial immunopotentiator OK-432 induced natural killer cell activating factor (NKAF) from mouse spleen cells. OK-432-induced NKAF showed a single peak with an apparent mol. wt of 70 Kd by Sephadex G-100 chromatography and OK-432-induced interleukin 2 (IL-2) had the same mol. wt as NKAF. However, OK-432-induced interferon (IFN) showed molecular heterogeneity with two peaks at 90 Kd and 45 Kd. Further purification was achieved by Blue Sepharose affinity chromatography which copurified NKAF and IFN. The affinity-purified NKAF, however, was stable to heat (56 degrees C) and acid (pH 2) treatments. Moreover, anti-IFN failed to abolish NKAF activity and this activity was not absorbed by IL-2 dependent T cells. From isoelectric focusing analysis, a dissociation of NKAF and IFN was observed over the range of pI 6.5 to 8.0. Based on these results, KNAF appears to be a new kind of cytokine distinguishable from IFN and IL-2.


Br. J. Cancer (1984), **50**, 97–108

### Characterization of mouse natural killer cell activating factor (NKAF) induced by OK-432: Evidence for interferon- and interleukin 2-independent NK cell activation

O. Ichimura[1], S. Suzuki[1], Y. Sugawara[1] & T. Osawa[2]

[1]*Research Laboratories I, Chugai Pharmaceutical Co., Ltd. and* [2]*Division of Chemical Toxicology and Immunochemistry, Faculty of Pharmaceutical Sciences, University of Tokyo, Tokyo, Japan.*

**Summary** The bacterial immunopotentiator OK-432 induced natural killer cell activating factor (NKAF) from mouse spleen cells. OK-432-induced NKAF showed a single peak with an apparent mol. wt of 70 Kd by Sephadex G-100 chromatography and OK-432-induced interleukin 2 (IL-2) had the same mol. wt as NKAF. However, OK-432-induced interferon (IFN) showed molecular heterogeneity with two peaks at 90 Kd and 45 Kd. Further purification was achieved by Blue Sepharose affinity chromatography which copurified NKAF and IFN. The affinity-purified NKAF, however, was stable to heat (56°C) and acid (pH 2) treatments. Moreover, anti-IFN failed to abolish NKAF activity and this activity was not absorbed by IL-2 dependent T cells. From isoelectric focusing analysis, a dissociation of NKAF and IFN was observed over the range of pI 6.5 to 8.0. Based on these results, KNAF appears to be a new kind of cytokine distinguishable from IFN and IL-2.

Lymphoid cells from many mammalian species exhibit spontaneous cell-mediated cytotoxicity against a variety of syngeneic, allogeneic and xenogeneic tumour target cells. The effector cells mediating spontaneous cytotoxicity have been designated natural killer (NK) cells, because of their natural tumoricidal capacity without any apparent presensitization (Pross *et al.*, 1977). There is strong evidence that NK cells infiltrate the tumour site (Flannery *et al.*, 1981; Moore & Moore, 1979) and play a role in protection against tumour growth (Habu *et al.*, 1981), and against metastasis of transplantable tumours (Hanna, 1980; Talmadge *et al.*, 1980). NK cells also have the ability to kill freshly isolated autologous tumour cells (Uchida *et al.*, 1983*b*). Recently, substantial interest has been generated in soluble factors and agents which influence the level of NK cell-mediated cytotoxicity. Murine NK activity has been shown to be enhanced by a variety of immunoregulating substances such as interferon (Gidlund *et al.*, 1978), interleukin 2 (Henney *et al.*, 1981) and thymic factor (Dominique *et al.*, 1983).

OK-432 is the one of the biological response modifiers which has been extensively used for malignant diseases. This agent has been found to enhance NK activity both in humans (Uchida & Mickshe, 1981) and in mice (Oshimi *et al.*, 1980). OK-432 also induces interleukin 2 (IL-2) and interferon (IFN) from human peripheral blood lymphocytes (Wakasugi *et al.*, 1982) and IFN from mouse spleen cells (Saito *et al.*, 1982). In the murine system, however, spleen cell-derived NK cell activating factor induced by OK-432 stimulation has not been fully characterized. In order to investigate the factor most responsible for NK cell activation by OK-432, we have studied the culture supernatants of OK-432-stimulated mouse spleen cells. As we report here, a soluble factor which demonstrates properties distinct from IFN and IL-2 plays an essential role in NK cell activation by OK-432.

### Materials and methods

### *Mice and tumour cells*

Specific pathogen free BALB/c mice (6 to 8 week-old female) were obtained from Charles River Japan, Inc. (Atugi, Japan). YAC-1 lymphoma cells were used as NK target cells and L929 cells were used for IFN assay. YAC-1 cells were maintained in RPMI 1640 medium (GIBCO, Grand Island, NY) supplemented with 10% heat-inactivated foetal calf serum (FCS, GIBCO) and 60 $\mu g\,ml^{-1}$ kanamycin (Meiji Seika Co., Ltd., Tokyo). L929 cells were cultured in minimum essential medium (MEM, Nissui Co., Ltd., Tokyo) supplemented with 5% FCS.

### *Stimulants*

OK-432, a penicillin- and heat-treated, lyophilized preparation of the low virulence Su strain of

Correspondence: O. Ichimura, Research Laboratories I, Chugai Pharmaceutical Co., Ltd., 3-41-8 Takada, Toshima-ku, Tokyo 171, Japan.
Received 27 February 1984; accepted 29 March 1984.

© The Macmillan Press Ltd., 1984

*Streptococcus pyogenes* group A3 (Chugai Pharmaceutical Co., Ltd., Tokyo, Japan), with KE (Klinische Einheit) unit corresponding to 0.1 mg dried streptococci, was suspended in physiological saline for *in vivo* administration and in RPMI medium for *in vitro* use. Concanavalin A (Con A) was purchased from Sigma Chemical Company (Saint Louis, MO).

*Preparation of NKAF containing splenocyte culture supernatants for gel filtration*

Spleens were obtained from 400 mice 4 days after i.p. injection of OK-432 (1 KE/mouse). The single cell suspension was prepared by pressing the tissue through a fine mesh screen into MEM. Splenocytes were washed twice with MEM and suspended at a concentration of $10^7$ cells ml$^{-1}$ in RPMI 1640 medium supplemented with 1% FCS (1% FCS-RPMI). The cell suspension was distributed in 30 ml aliquots into 75 cm$^2$ plastic culture flasks (Corning Glass Works, Corning, NY) and incubated with OK-432 (0.05 KE ml$^{-1}$) for 24 h at 37°C in a humidified atmosphere containing 5% $CO_2$. Then the culture supernatants were collected by centrifugation at 220 *g* for 10 min. To remove OK-432 particles, an additional centrifugation at 12,000 *g* for 30 min was performed.

*Sephadex G-100 chromatography*

Cell free supernatants were precipitated by 80% ammonium sulphate at 4°C for 24 h. After centrifugation at 12,000 *g* for 30 min, the pellets were redissolved in 0.1 M PBS (pH 7.4) and dialyzed against the same buffer overnight at 4°C, then concentrated 100-fold from the starting supernatant volume by polyethylene glycol 20,000 (Wako Pure Chemical Ind., Osaka, Japan). Fifteen mililiters of concentrated materials (44 mg ml$^{-1}$) were dialyzed against running buffer (0.1 M PBS, pH 7.4 containing 0.5 M NaCl and 0.02% $NaN_3$), applied onto the Sephadex G-100 column (6 × 150 cm) and eluted in 14 ml fractions at a flow rate of 57.1 ml h$^{-1}$. Then, 1 ml of each fraction was dialyzed against RPMI 1640 medium overnight, filtered and assayed for NKAF activity. Apparent mol. wt was estimated by comparison with the standard proteins: blue dextran (200 Kd), bovine serum albumin (BSA, 67 Kd), ovalbumin (OVA, 45 Kd), chymotrypsinogen (CHY, 23 Kd) and ribonuclease A (RIB, 13 Kd) [(Low Mol. Wt Calibration Kit (Pharmacia Fine Chemicals, Uppsala, Sweden))].

*Blue Sepharose CL-6B affinity chromatography*

Sephadex-active NKAF fractions (fr. 55–65) were collected and purified by affinity chromatography according to the methods of Stefanos *et al.* (1980). Briefly, Blue Sepharose CL-6B resin (Pharmacia Fine Chemicals) was washed with 100 column volumes of starting buffer E1 (0.02 M PBS, pH 7.4) and packed into a 12 × 61 mm column. Then 2 ml of active materials (38 mg ml$^{-1}$) dialyzed against E1 buffer was applied to the column and washed with 10 column vols of starting buffer E1. After washing, the absorbed materials were eluted with a discontinuous NaCl gradient: second buffer E2 (0.2 M NaCl), third buffer E3 (1.5 M NaCl) and final buffer E4 (50% v/v ethylene glycol in E3 buffer). The flow rate was 2.2 ml h$^{-1}$ and each fraction was 1.06 ml.

*Isoelectric focusing-polyacrylamide gel electrophoresis (IEF-PAGE)*

The pI of NKAF was determined by IEF-PAGE. The gels (5%, 80 mm) contained 2% ampholytes (pH 3–10, Bio-Rad Laboratories, Richmond, CA) and were polymerized with riboflavin-5-phosphate ($5 \times 10^{-6}$%), ammonium persulfate ($2 \times 10^{-4}$%) and with light for 3 h. Samples were dialyzed against distilled water, and then glycerol (15% v/v) and ampholytes (2% v/v) were added. Then, 100 μl aliquot of samples were applied to the gels and overlayered first with 100 μl of 5% glycerol and 2% ampholytes and second with upper buffer (0.02 M $H_2SO_4$). The lower buffer was 1 M NaOH. The gels were electrophoresed at a constant voltage of 200 v for 24 h at 4°C, removed and sliced into 3 mm sections, and placed in tubes containing degassed distilled water. The tubes were tightly capped and incubated overnight at 4°C to elute NKAF. The pH of each sample was measured and eluants were dialyzed against distilled water overnight at 4°C. Then all samples were lyophilized, and 300 μl of RPMI 1640 medium added to each fraction.

*Determination of NKAF activity*

Splenocytes of BALB/c mice were suspended at a concentration of $1.35 \times 10^7$ cells ml$^{-1}$ in 10% FCS-RPMI medium. An aliquot (75 μl) of effector cell suspension was placed into a 96 well round bottom microplate (Nunclon, Nunc Inter Med., Denmark). Effector cells were preincubated with NKAF containing samples (25% v/v) at 37°C for 3 h. After preincubation, $^{51}$Cr-labelled YAC-1 target cell suspension (100 μl well$^{-1}$) was added to each well. The cells were incubated at 37°C for an additional 4 h. Then 100 μl of supernatant was removed and the radioactivity determined in a gamma counter. Spontaneous release was determined for 100 μl of target cells incubated in medium alone and total incorporated cpm were determined for 100 $\mu$l of target cell suspension. The percentage of specific cytolysis was calculated using the following formula:

% specific cytolysis

$$= \frac{\text{Experimental cpm} - \text{Spontaneous cpm}}{\text{Total Incorporated cpm} - \text{Spontaneous cpm}} \times 100$$

NKAF activity was assayed in triplicate.

*Determination of IFN activity*

IFN activity was determined by using the 50% plaque reduction method on mouse L929 cells with vesicular stomatitis virus added to the microculture plate as described by Koi *et al.* (1981). IFN activity was expressed in international reference units based on an NIH reference mouse IFN (NIH No. G-002-904-511). All samples were assayed in duplicate.

*Determination of IL-2 activity*

The IL-2 dependent allogeneic killer T cell line, Clone 902, which was established from mixed lymphocyte cultures of A/J mouse spleen cells immunized with C57BL/6 mouse spleen cells, had been maintained in our laboratories over 6 months in the presence of rat IL-2. The IL-2 dependent cells were washed 3× with MEM prior to IL-2 assay and suspended in 5% FCS-RPMI medium and placed in a 96 well round bottom microculture plate ($10^4$ cells/well); then, IL-2 containing samples were added to the microculture at a concentration of 10% (v/v) in 200 $\mu$l of final volume. Microcultures were incubated with 1.0 $\mu$Ci $^3$H-thymidine for the last 4 h of the 48 h incubation and were harvested on glass fibre filters by using the LABO MASH semi-automatic cell harvester (Labo Science, Tokyo, Japan). Incorporation of radioactivity was determined by scintillation counting in a toluene base scintillator. IL-2 activity was expressed as the mean incorporated cpm of triplicate assay.

*IL-2 absorption assay*

IL-2 dependent cells were washed 3× with IL-2 free MEM to remove remaining IL-2. Then the cell pellets ($10^4$–$10^7$ cells) were incubated in test sample solution (1 ml) at 4°C for 4 h to allow absorption. The supernatants were removed by centrifugation at 220 *g* for 5 min and bioassayed for NKAF and IL-2 activity.

*Heat treatment*

Samples were incubated at 56°C for 30 min or 60 min in a water bath to compare the stability of NKAF and IFN. After incubation, heat-treated materials were immediately cooled in ice for later use.

*Anti-IFN serum and IFN*

Anti-IFN $\alpha/\beta$ (NIH No. G024-501-568) was kindly provided by Dr K. Pauker, Medical College of Pennsylvania, Philadelphia, PA. Anti-IFN$\gamma$, a rabbit antiserum against mouse lymphocyte IFN which is induced by *Staphyloccoccal enterotoxin* A (Langford *et al.*, 1981) was supplied by Dr M.P. Langford, University of Texas, Galveston, TX. L cell-derived IFN (specific activity $2.2 \times 10^8$ units ml$^{-1}$, Torey Industries, Inc., Japan) was a kind gift of Dr N. Ishida, Tohoku University School of Medicine, Sendai, Japan.

*Acid treatment*

The samples were dialyzed against 0.01 M glycine-HCl pH 2 buffer at 4°C for 24 h to inactivate IFN$\gamma$. To neutralize to pH 7.2, an additional dialysis was performed against RPMI 1640 medium at 4°C overnight.

### Results

*Induction of NKAF from OK-432-stimulated mouse spleen cells*

Spleen cells of BALB/c mice injected with OK-432 (1 KE/mouse, i.p.) were isolated 1–7 days after injection and stimulated with OK-432 or Con A. Each 24 h culture supernatant was assayed for NKAF activity. As shown in Figure 1, untreated mouse spleen cells produced NKAF in response to stimulation by OK-432. However, from days 3–5, spleen cells of OK-432 treated mice showed levels of NKAF production higher than did untreated mouse spleen cells. The maximum NKAF production was observed in the supernatant of Day-4 spleen cells. In contrast to OK-432 stimulation, Con A failed to elicit NK-enhancing substances from OK-432 injected mouse spleen cells at levels higher than from untreated mouse spleen cells.

*Induction kinetics of NKAF*

In order to compare the induction kinetics of NKAF with those of IFN, spleen cell suspensions of untreated or OK-432 injected (Day-4) mice ($10^7$ cells ml$^{-1}$ in 1% FCS-RPMI medium) were incubated with OK-432 (0.05 KE ml$^{-1}$) at 37°C for 24 h, then each culture supernatant was assessed for NKAF and IFN activity. NKAF activity was detectable as early as 1 h after OK-432 stimulation and maximum NKAF production was observed in the culture supernatant of Day-4 splenocytes after 24 h incubation. The splenocytes of untreated mice

**Figure 1** Augmentation of NKAF production by OK-432 administration. Spleen cells of BALB/c mice given an i.p. injection of OK-432 (1 KE/mouse) after 1–7 days were stimulated with OK-432 (0.05 KE $ml^{-1}$, ▨), Con A (5 $\mu g\,ml^{-1}$, ▦) or medium alone (□) for 24 h. Each culture supernatant was assayed for NKAF activity at an E:T ratio of 50:1. Values represent mean ± s.d. of triplicate assay.

showed kinetically different NKAF production from that of Day-4 splenocytes, because untreated mouse splenocytes demonstrated levels of NKAF production at 24 h of cultivation lower than those of 4-day splenocytes and higher levels of NKAF production after 48 h incubation. In contrast to NKAF production, both splenocyte preparations showed the same IFN production kinetics. IFN activity was detectable at 7 h after OK-432 stimulation, reached a plateau after 12 h and remained unchanged for at least 48 h (Figure 2).

*Dose-dependent NKAF production in response to OK-432 stimulation*

To investigate the dose-dependency of NKAF production in response to OK-432 stimulation *in vitro*, the untreated mouse splenocytes and Day-4 splenocytes ($10^7$ cells $ml^{-1}$ in 1% FCS-RPMI medium) were incubated with OK-432 ($5\times10^{-4}$, $5\times10^{-3}$ or $5\times10^{-2}$ KE $ml^{-1}$) or medium alone for 24 h. As shown in Figure 3, Day 4 splenocytes showed levels of NKAF production higher than untreated mouse splenocytes at the concentration of $5\times10^{-3}$ and $5\times10^{-2}$ KE $ml^{-1}$. However, both splenocyte populations demonstrated dose-dependent NKAF production in response to

**Figure 2** Induction kinetics of NKAF and IFN. Spleen cells of untreated mice and Day-4 mice were incubated with OK-432 (0.05 KE $ml^{-1}$) for 1–48 h. Each culture supernatant was assayed for NKAF and IFN activity. NK activity in the presence of culture supernatants of untreated mice spleen cells (○), Day-4 mice spleen cells (●) or medium alone (□). Each value represents mean ± s.d. of triplicate assay at an E:T ratio of 50:1. IFN activity of untreated (□) or Day-4 mice (▨) culture supernatants.

**Figure 3** OK-432 dose-dependent NKAF production by spleen cells. NK activity in the presence of culture supernatants of untreated mice (○), Day-4 mice (●) splenocytes incubated with various concentrations of OK-432 ($5\times10^{-4}$ to $5\times10^{-2}$ KE ml$^{-1}$) or medium alone (□). Each value represents mean ± s.d. of triplicate assay at an E:T ratio of 50:1. IFN activity of untreated mice (□) and Day-4 mice (▨) splenocytes culture supernatants.

**Figure 4** Augmentation kinetics of NK activity by NKAF-containing culture supernatants. Fresh spleen cells were incubated with NKAF containing culture supernatants (Day-4 spleen cell-derived, with 1020 units ml$^{-1}$ IFN activity, (●), IFN (□) or medium alone (○) for 0–20 h prior to NKAF assay. Each value represents mean ± s.d. triplicate assay at an E:T ratio of 50:1.

the concentration of OK-432. In contrast to NKAF production, there was not a significant difference in IFN production by each splenocyte population: IFN activity was detectable at a concentration of $5\times10^{-3}$ KE ml$^{-1}$ and reached a plateau at a concentration of $5\times10^{-2}$ KE ml$^{-1}$. Although repeated injections of OK-432 (1 KE/mouse, i.p.) enhanced IFN production by spleen cells following stimulation of OK-432 or Con A *in vitro* (Saito *et al.*, 1983), a single injection of OK-432 failed to enhance IFN production. Nevertheless, a single injection of OK-432 enhanced NKAF production to the rechallenge of OK-432 *in vitro*.

### *Augmentation kinetics of NK activity by NKAF containing culture supernatants*

In order to investigate the augmentation kinetics of NK activity by NKAF, 24 h culture supernatants of Day-4 splenocytes stimulated with OK-432 (0.05 KE ml$^{-1}$) concentrated by 80% ammonium sulphate (2.2 mg ml$^{-1}$), or L cell-derived IFN (3,400 units ml$^{-1}$) were added to fresh splenocytes at 25% v/v, incubated at 37°C for 0 to 20 h, then assayed for NK activity. As shown in Figure 4, NKAF containing supernatants caused significant boosting of NK activity without preincubation. Maximum augmentation of NK activity was detected at 3 h preincubation. IFN-induced NK cell activation showed kinetics resembling those of NKAF containing supernatants; reaching a plateau at 3 h preincubation and slightly decreasing at 20 h.

### *Sephadex G-100 chromatography of NKAF*

In order to investigate the mol. wt of OK-432 induced NKAF, culture supernatants from Day 4 spleen cells stimulated with OK-432 (0.05 KE ml$^{-1}$, 24 h), conditions which demonstrated maximum NKAF production, were concentrated and chromatographed on a Sephadex G-100 column. A major peak of NKAF was observed at an apparent mol. wt of ~70 Kd coinciding with the mol. wt of OK-432-induced IL-2. However, OK-432-induced IFN showed molecular heterogeneity: two peaks at ~80 Kd and 45 Kd. From the results of gel filtration, the mol. wt of NKAF correlated well with IL-2 but not with IFN (Figure 5).

### *Failure of NKAF to be absorbed by IL-2 dependent cells*

By Sephadex G-100 chromatography, NKAF and OK-432-induced IL-2 were known to have a closely related mol. wt of 70 Kd. To investigate the relationship of NKAF and IL-2, the Sephadex

**Figure 5** Sephadex G-100 chromatography of OK-432-induced NKAF. Culture supernatants of Day-4 splenocytes stimulated with OK-432 (0.05 KE ml$^{-1}$) for 24 h were prepared for chromatography on Sephadex G-100 as described in **Materials and methods**. Each fraction was assayed for NKAF activity (●), IFN activity (▨) and IL-2 activity (○). NK activity incubated with medium alone was 11.5 ± 1.7 ⊞.

active NKAF fractions (fr. 55–65) were collected and dialyzed against RPMI medium. Then the active materials were incubated with IL-2 dependent cells ($10^4$–$10^7$ cells ml$^{-1}$) at 4°C for 4 h. After incubation, each supernatant was assayed for NKAF and IL-2 activity. As shown in Table I, NKAF activity remained unchanged even after incubation with IL-2 dependent cells. Before IL-2 absorption, the materials did not demonstrate any significant IL-2 activity in spite of the IL-2 peak fraction obtained by gel filtration. We observed that OK-432-induced IL-2 showed 10 or more times less activity than Con A-induced IL-2 in unseparated culture supernatants (Ichimura *et al.*, 1983) and was liable to concentration and dialysis procedures (data not shown). Nevertheless NKAF activity was present in concentrate materials and was not absorbed by IL-2 dependent cells.

*Blue Sepharose CL-6B affinity chromatography of Sephadex active NKAF*

IFNγ can be selectively purified by Blue Sepharose affinity chromatography (Stefanos *et al.*, 1980). In order to remove IFNγ from NKAF containing materials, Sephadex active NKAF fractions (fr. 55–65) were pooled, concentrated (38 mg ml$^{-1}$), and loaded on to an affinity column. As shown in Figure 6, NKAF and IFN were eluted in the same fraction by E3 buffer (1.5 M NaCl containing PBS). IL-2 activity was not observed in any fractions.

*Stabilities of affinity-purified NKAF and the anti-IFN serum treatment*

To compare the physicochemical properties of NKAF and IFN, the affinity-purified NKAF fraction (fraction E3) was treated with heat (56°C, 30 min or 60 min) and acid (pH 2, 24 h). As shown in Table II, NKAF activity remained unchanged after either treatment; however, IFN was sensitive to both heat and acid treatments. Nevertheless, high titres of IFN activity still remained after acid and heat treatment (320 units ml$^{-1}$ and 640 units ml$^{-1}$ respectively). It is possible that residual IFN affects NK cell activity. In order to investigate this possibility, anti-IFN serum was added to the acid-treated materials to neutralise residual IFN activity. Although anti-IFNγ completely neutralized IFN activity, it failed to abolish NKAF activity. Anti-IFN α/β did not neutralize IFN activity but reduced NKAF activity to the same extent as anti-IFNγ. Since the partial reduction of NKAF activity by anti-IFN serum was not correlated with neutralization of IFN activity, NKAF was distinguishable from IFN by its antigenicity.

*Effect of NKAF presence or absence in cytotoxic assay*

In order to investigate whether or not the presence of NKAF in the cytotoxic assay is a pre-requisite for augmentation of NK activity, spleen cells were incubated with affinity-purified NKAF at 25% v/v for 3 h, washed once and assessed for NK activity. As shown in Table III, the presence of NKAF throughout the cytotoxic assay resulted in a higher level of NK-boosting activity than the absence of NKAF. However, an appreciable level of NK-boosting activity remained after the washing out of NKAF prior to the cytotoxic assay.

**Table I** Failure of IL-2 dependent cells to absorb NKAF

| *Absorbed CTLL cell number* | *Residual NKAF activity*[a] *(% specific killing)* | *Residual IL-2 activity*[b] *(incorporated cpm)* |
|---|---|---|
| $10^7$ | 22.2 ± 0.67 | 94 ± 22[c] |
| $5 \times 10^6$ | 24.6 ± 1.05 | 54 ± 6[c] |
| $10^6$ | 21.4 ± 2.33 | 59 ± 5[c] |
| $5 \times 10^5$ | 21.1 ± 2.95 | 59 ± 3[c] |
| $10^5$ | 26.6 ± 1.89 | 76 ± 15[c] |
| $5 \times 10^4$ | 25.3 ± 0.35 | 70 ± 7[c] |
| $10^4$ | 24.6 ± 1.48 | 84 ± 13[c] |
| 0 | 20.9 ± 1.92 | 58 ± 17[c] |
| Medium alone | 7.8 ± 0.99 | 112 ± 25 |

Sephadex G-100 active NKAF fraction (fr. 55–65) was incubated with IL-2 dependent T cells at 4°C for 4 h.

[a]Each value represents mean ± s.d. of triplicate assay at an E:T ratio of 50:1.

[b]Each value represents mean ± s.d. of triplicate assay.

[c]Value was insignificant from medium control.

**Figure 6** Blue Sepharose affinity chromatography of Sephadex G-100 active NKAF fraction. Sephadex G-100 active NKAF fraction (fr. 55–65) was prepared for chromatography on Blue Sepharose CL-6B. Absorbed materials were eluted with a discontinuous NaCl gradient (E1 buffer, 0.02 M PBS, pH 7.4; E2 buffer, 0.2 M NaCl PBS; E3 buffer, 1.5 M NaCl PBS; E4 buffer, 50% v/v ethylene glycol in E3 buffer). Each fraction was assayed for NKAF activity (●) and IFN activity (○). NK activity incubated with medium alone was 10.8 ± 0.7 ▦.

**Table II** Stabilities and anti-IFN serum treatments of affinity-purified NKAF

| *Treatment*[a] | *Residual NKAF activity*[b] (*% specific killing*) | *Residual IFN activity* (*inits ml*$^{-1}$) |
|---|---|---|
| Untreated | 11.1 ± 0.36 | 1280 |
| Heat 56°C, 30 min | 12.1 ± 0.95 | 640 |
| 60 min | 10.4 ± 0.17 | 640 |
| Acid pH 2, 24 h | 10.4 ± 0.14 | 320 |
| + anti-IFN $\alpha/\beta$[c] | 8.6 ± 1.27 | 320 |
| + anti-IFN $\gamma$[d] | 7.5 ± 1.27 | < 20 |
| Medium alone | 0.2 ± 0.58 | — |

[a]Blue Sepharose active NKAF fraction E3 was treated.

[b]Each value represents mean ± s.d. of triplicate assay at an E:T ratio of 50:1.

[c]Anti-IFN $\alpha/\beta$ was equivalent to 100,000 neutralizing units ml$^{-1}$.

[d]Anti-IFN $\gamma$ was equivalent to 320 neutralizing units ml$^{-1}$.

**Table III** NKAF presence or absence in cytotoxic assay

| | *NK activity (% specific lysis)*[a] | | |
|---|---|---|---|
| | *Medium alone* | *NKAF presence*[b] | *NKAF absence*[c] |
| Exp. 1[d] | | | |
| 3 h preincubation | 1.5±0.07 | 12.7±0.43 | 7.1±0.38 |
| Exp. 2[e] | | | |
| 24 h preincubation | 4.8±0.89 | 23.1±0.98 | 17.0±0.41 |

[a]Each value represents mean ± s.d. of triplicate assay.
[b]Blue Sepharose partially purified NKAF (0.42 mg ml$^{-1}$ with 1020 units ml$^{-1}$ of IFN activity) present in cytotoxic assay after 3 h preincubation.
[c]Preincubated cells were washed once prior to NK assay.
[d]E:T ratio was 33:1.
[e]E:T ratio was 50:1.

*Augmentation of splenic and lymphatic NK activity but not thymic NK activity by NKAF*

In order to elucidate the organ distribution of NKAF-reactive effector cells other than splenocytes, thymocytes and mesenteric lymph node cells were used as effector cells, preincubated with affinity-purified NKAF fr. E3 at various NKAF concentrations for 3 h and assessed for NK activity. As shown in Figure 7, thymocytes failed to react with NKAF but NK activity of mesenteric lymph node cells as well as splenocytes were augmented by NKAF in a dose-dependent fashion. These findings show the lack of NKAF-reactive effector cells in thymus but not in mesenteric lymph node and spleen.

**Figure 7** *In vitro* augmentation of splenic and lymph node NK activity but not thymic NK activity by NKAF. Spleen, mesenteric lymph node and thymus cells were incubated with affinity purified NKAF (0.42 mg ml$^{-1}$, with 1020 units ml$^{-1}$ of IFN activity) at various diluted concentrations as indicated in figure for 3 h prior to NK assay. NK activity of spleen (●), mesenteric lymph node (▲) and thymus (■) cells were expressed as mean ± s.d. of triplicate assay at an E:T ratio of 33:1.

*Isoelectric focusing (IEF)-PAGE of affinity-purified NKAF*

Because IEF possesses a relatively high degree of resolving power for the separation of proteins of similar, but not identical charge, it has been quite useful in comparative studies of the biological activities present in the same cell culture supernatants. It was of interest to compere the pI of NKAF and IFNγ. The affinity-purified NKAF fraction was first dialyzed against distilled water, a procedure which did not significantly affect NKAF activity (data not shown). The dialyzed materials were then electrophoresed for 18 h at a constant voltage of 200 v at 4°C. As shown in Figure 8, NKAF showed charge heterogeneity, with two major peaks (pI 4.0–6.0 and pI 6.5–8.0). Affinity-purified IFN, however, had an acidic pI 4.0–6.0 only. These findings demonstrate that NKAF could be partially separated from IFN by IEF-PAGE.

### Discussion

We found that an IL-2 and IFN-independent pathway in the process of NK cell activation by OK-432 exists which is mediated by a new kind of soluble factor termed NKAF. This conclusion was based on the results from the following

**Figure 8** Isoelectric focusing PAGE of affinity-purified NKAF. Affinity-purified NKAF (fr. E3) was electrophoresed by IEF-PAGE as described in **Materials and methods**. Each fraction was assayed for NKAF activity (●) at an E:T ratio of 50:1 and for IFN activity (○). NK activity incubated with medium alone was 10.7 ± 1.5.

experiments: (i) the induction kinetics and the optimal concentration of OK-432 for the production of NKAF were different from those of IFN (Figures 2, 3) and single i.p. injection of OK-432 caused enhancement of NKAF production but not IFN in response to *in vitro* OK-432 stimulation (Figures 2, 3). (ii) NKAF and OK-432-induced IL-2 demonstrated the same mol. wt 70 Kd, however, OK-432-induced IFN showed molecular heterogeneity by gel filtration analysis (Figure 5). (iii) Although affinity-purified IFN was labile to heat (56°C) and acid (pH 2) treatments, NKAF was not affected by these treatments. Moreover, an additional anti-IFN antiserum treatment failed to abolish NKAF activity (Table II). (iv) NKAF demonstrated charge heterogeneity in IEF-PAGE experiments. Furthermore, a dissociation of NKAF and IFN was observed over the pI range of 6.5 to 8.0 (Figure 8).

In the human system, Uchida *et al.* (1981) reported IL-2- and IFN-independent NK cell activation by OK-432. Their finding that anti-IFN serum failed to inhibit NK cell activation by OK-432 correlated well with our observations. In contrast to our present results, Wakasugi *et al.* (1982) reported the participation of IL-2 and IFN in NK cell activation by OK-432: IL-2, IFNα and IFNγ were induced in culture supernatants of OK-432-stimulated human peripheral lymphocytes. These discrepancies may result from differences in species, in the preparation of OK-432-stimulated culture supernatants, or in the time of pretreatment of effector cells (24 h). We used the culture supernatant of OK-432-pretreated (Day-4) mouse splenocytes as a source of NKAF and employed a short term preincubation (3 h) system. According to Henney *et al.* (1981), IL-2-induced boosting of NK activity is observed after 16 h of culture. More than 6 h of preincubation is required for recombinant IFNγ-induced NK cell activation (John R. Ortaldo, personal communication) but not for recombinant IFNα, a protein which causes augmentation of NK activity, within hours (Herberman *et al.,* 1982). OK-432-induced IFN, copurified with NKAF, was gamma type because of its acid susceptibility and antigenicity (Table II). These findings suggest that contaminating IFNγ and IL-2 do not affect NKAF activity in a short term preincubation system; even so, we could not dismiss the possibility that contaminating IFNγ and IL-2 may have altered the sensitivity of our effector cells to NKAF.

Although the cellular origin of NKAF remains unclear, our recent studies suggest that thymus-derived lymphocytes may be the NKAF producer cells, because thymocytes produce NKAF-like factor in response to OK-432 stimulation *in vitro*. This factor resembles spleen-cell derived NKAF in terms of its chromatographic behaviour, but differs in terms of its antigenicity to IFN and IL-2, among other characteristics. I.p. administration of OK-432 prior to *in vitro* restimulation by OK-432 failed to augment IFN production but did augment NKAF

production. These observations led us to suggest that a single injection of OK-432 is sufficient for NKAF induction but not for IFN induction and, furthermore, that the induction mechanisms might be different from each other. As previously reported, a single i.p. injection of OK-432 enhanced both interleukin 1 (IL-1) and IL-2 production in response to the *in vitro* rechallenge of OK-432 (Ichimura *et al.*, 1983). These interleukins may participate in NKAF production, since IL-1 augments IL-2 production and IL-2 regulates IFN$\gamma$ production by T cells (Farrar *et al.*, 1982).

The presence of NKAF throughout the cytotoxic assay resulted in higher levels of NK augmentation than did the absence of NKAF even after 24 h of preincubation (Table III). NKAF did not demonstrate any direct cytotoxicity against YAC-1 target cells in a 4 h chromium release assay (data not shown). These observations suggest that NKAF might affect not only NKAF-responsive effector cells but also effector to target interaction or target susceptibility to NK cell-mediated cytolysis.

NKAF preparations augmented splenic and mesenteric lymph node NK cell activity in a dose dependent manner but not thymic NK cell activity (Figure 7). In the human system, OK-432 can activate peripheral blood and lymph node NK cell activity by *in vitro* stimulation. However, IFN failed to activate lymph node NK cell activity (Uchida *et al.*, 1984). Our preliminary observation that murine NKAF enhanced human peripheral blood NK cell activity (data not shown) led us to the speculation that the mechanisms of NKAF-induced NK cell activation might be different from those of IFN-induced NK cell activation which is a well documented species-specific phenomenon (Heron *et al.*, 1979; Herberman *et al.*, 1982).

In human cancer patients, intrapleural administration of OK-432 resulted in an induction or augmentation of effusion NK cell activity along with reduction or disappearance of effusion tumour cells. Autologous tumour killer cells induced by OK-432 demonstrated NK cell morphological characteristics (Uchida *et al.*, 1983*a*). These clinical findings suggest that NK cell activation is most responsible for the anti-tumour effect of OK-432.

In conclusion, our observations indicate that the regulation of mouse NK activity *in vitro* is complex and subject to the participation of NKAF as well as IFN and IL-2 in OK-432 stimulation. These findings provide the basis for future studies on the modulation of NK activity.

We thank Dr N. Ishida for kindly supplying antiserum against mouse IFN. We also wish to thank Dr M. Saito for his technical advice and Dr A. Uchida for helpful suggestions.

**References**

DOMINIQUE, K., PRIEUR, D. & STARKEY, J. (1983). *In vitro* modulation of natural killer (NK) activity by serum thymic factor (FTS). *Cell. Immunol.*, **76**, 232.

FARRAR, J.J., BENJAMIN, W.R., HILFIKER, M.L., HOWARD, M., FARRAR, W.L. & FULLER-FARRAR, J. (1982). The biochemistry, biology, and role of interleukin 2 in the induction of cytotoxic T cells and antibody-forming B cell responses. *Immunol. Rev.*, **63**, 12.

FLANNEY, G.R., ROBINS, R.A. & BALDWIN, R.W. (1981). Natural killer cells infiltrate transplanted chemically induced sarcoma. *Cell. Immunol.*, **61**, 1.

GIDLUND, M., ÖRN, A., WIGZELL, H., SENIK, A. & GRESSER, I. (1978). Enhanced NK activity in mice injected with interferon and interferon inducers. *Nature*, **273**, 759.

HABU, S., FUKUI, H., SHIMAMURA, K. & 4 others. (1981). *In vivo* effects of anti-asialo GM1. I. Reduction of NK activity and enhancement of transplanted tumor growth in nude mice. *J. Immunol.*, **127**, 34.

HANNA, N. (1980). Expression of metastatic potential of tumor cells in young nude mice is correlated with low level of natural killer cell-mediated cytotoxicity. *Int. J. Cancer*, **26**, 675.

HENNEY, C.S., KURIBAYASHI, K., KERN, D.E. & GILLIS, S. (1981). Interleukin 2 augments natural killer cell activity. *Nature*, **291**, 335.

HERBERMAN, R.B., ORTALDO, J.R., MANTOVANI, A., HOBBS, D.S., KUNG, H.-F. & PESTKA, S. (1982). Effect of human recombinant interferon on cytotoxic activity of natural killer (NK) cell and monocytes. *Cell. Immunol.*, **67**, 160.

HERON, I., HOKLAND, M., MØLLER-LARSEN, A. & BERG, K. (1979). The effect of interferon on lymphocyte-mediated effector cell: selective enhancement of natural killer cells. *Cell. Immunol.*, **42**, 183.

ICHIMURA, O., SUZUKI, S., SUGAWARA, Y. & OSAWA, T. (1983). Lymphokines induction by Streptococcal preparation OK-432 (Picibanil) in mice: Characterization of interleukin (IL-1), interleukin 2 (IL-2) and natural killer cell activating factor (NKAF). In: *Proceedings the 13th International Congress of Chemotherapy*, (Eds. Spitzy & Karrer), Vienna: V.H. Egermann (in press).

KOI, M., SAITO, M., EBINA, T. & ISHIDA, N. (1981). Lactate dehydrogenase-elevating agent is responsible for interferon induction and enhancement of natural killer cell activity by inoculation of Ehrlich ascites carcinoma cells into mice. *Microbiol. Immunol.*, **25**, 565.

LANGFORD, M., WEIGENT, D.A., GEORGIADES, J.A., JOHNSON, H.M. & STANTON, G.J. (1981). Antibody to staphylococcal enterotoxin A-induced human immune interferon (IFN$\gamma$). *J. Immunol.*, **126**, 1620.

MOORE, K. & MOORE, M. (1979). Systemic and *in-situ* natural killer activity in tumour-bearing rats. *Br. J. Cancer*, **39**, 636.

OSHIMI, K., KANO, S., TAKAKU, F. & OKUMURA, K. (1980). Augmentation of mouse natural killer cell activity by a streptococcal preparation, OK-432. *J. Natl Cancer Inst.*, **65**, 1265.

PROSS, H.F. & BAINES, G.M. (1977). Spontaneous human lymphocyte-mediated cytotoxicity against tumor target cells. VI. A brief review. *Cancer Immunol. Immunother.*, **3**, 75.

SAITO, M., EBINA, T., KOI, M., YAMAGUCHI, T., KAWADE, Y. & ISHIDA, N. (1982). *In vitro* production of immune interferon (IFN$\gamma$) in mouse spleen cells by OK-432, a preparation of *Streptococcus pyogenes*. *Cell. Immunol.*, **68**, 187.

SAITO, M., YAMAGUCHI, T., EBINA, T. & 4 others. (1983). *In vitro* production of immune interferon (IFN$\gamma$) by murine spleen cells when different sensitizing agents are used *in vivo* and *in vitro*. *Cell. Immunol.*, **78**, 379.

STEFANOS, S., CATINOT, L., WIETZERBIN, J. & FALCOFF, E. (1980). production of antibodies against mouse immune interferon and their neutralizing properties. *J. Gen. Virol.*, **50**, 225.

TALMADGE, J., MEYERS, K., PRIEUR, D. & STARKEY, J. (1980). Role of NK cells in tumor growth and metastasis in beige mice. *Nature*, **284**, 622.

UCHIDA, A. & MICKSHE, M. (1981). *In vitro* augmentation of natural killing activity by OK-432. *Int. J. Immunopharmacol.*, **3**, 365.

UCHIDA, A. & MICKSHE, M. (1983*a*). Intrapleural administration of OK-432 in cancer patients: activation of NK cells and reduction of suppressor cells. *Int. J. Cancer*, **31**, 1.

UCHIDA, A. & MICKSHE, M. (1983*b*). Lysis of fresh human tumor cells by autologous large granular lymphocytes from peripheral blood and pleural effusions. *Int. J. Cancer*, **32**, 37.

UCHIDA, A., YAGITA, M. & HOSHINO, T. (1984). Augmentation of natural and auto-tumor killing activity by OK-432. In: *NK Activity and its Regulation*. (Eds. Hoshino *et al.*), Tokyo: Excerpta Medica, p. 220.

WAKASUGI, H., KASAHARA, T., MINATO, N., HAMURO, J., MIYATA, M. & MORIOKA, Y. (1982). *In vitro* potentiation of human natural killer cell activity by a Streptococcal preparation, OK-432: interferon and interleukin 2 participation in the stimulation with OK-432. *J. Natl Cancer Inst.*, **69**, 4.